# Supplementary material for: The role of carbon nanoparticle in lymph node detection and parathyroid gland protection during thyroidectomy for non-anaplastic thyroid carcinoma- a meta-analysis
Source: PLoS One. 2020 Nov 10;15(11):e0223627. doi: 10.1371/journal.pone.0223627 (PMC7654818; doi:10.1371/journal.pone.0223627)
Supplement: S1 File — (DOCX) [file pone.0223627.s002.docx]

**The full search strategy in pubmed database**

Date and time: 2018/12/01 19:00

#1: "thyroid gland"[MeSH Terms] OR ("thyroid"[All Fields] AND "gland"[All Fields]) OR "thyroid gland"[All Fields] OR "thyroid"[All Fields] OR "thyroid (usp)"[MeSH Terms] OR ("thyroid"[All Fields] AND "(usp)"[All Fields]) OR "thyroid (usp)"[All Fields]  **Items found** [**211695**](https://www.ncbi.nlm.nih.gov/pubmed/?cmd=HistorySearch&querykey=18)

#2: "carbon"[MeSH Terms] OR "carbon"[All Fields]  **Items found** [**474922**](https://www.ncbi.nlm.nih.gov/pubmed/?cmd=HistorySearch&querykey=30)

#3: #1 and #2 **Items found 1153**

**Table 1. Details of Quality Assessment of Randomized Controlled Trials (Jadad scale)**

| Study | Randomization | Concealment of Allocation | Blinding | Loss to Follow-up% | Quality Assessment |
| --- | --- | --- | --- | --- | --- |
| Chen, W (2014) | No detailed description | Only mentioned randomized | Unclear | 0 | 2 |
| Gao, B (2015) | No detailed description | Only mentioned randomized | Unclear | 0 | 2 |
| Gao, Q (2014) | No detailed description | Only mentioned randomized | Unclear | 4 | 3 |
| Gu, J(2015) | No detailed description | Only mentioned randomized | Unclear | 0 | 2 |
| Liu, F (2017) | No detailed description | Only mentioned randomized | Unclear | 0 | 2 |
| Long, M(2017) | Computer-generated sequence | Concealed envelopes | Single blinding | 14.6 | 5 |
| Tian, W(2014) | No detailed description | Only mentioned randomized | Unclear | 0 | 2 |
| Wang, B(2015) | Computer-generated sequence | Concealed envelopes | Single blinding | 0 | 5 |
| Xu, X. F (2017) | No detailed description | Only mentioned randomized | Unclear | 0 | 2 |
| Yu, W (2016). | Computer-generated random number tables | Only mentioned randomized | Single blinding | 0 | 4 |
| Yu, WB(2016) | Computer-generated random number tables | Only mentioned randomized | Single blinding | 0 | 4 |
| Zhu, H (2017) | No detailed description | Only mentioned randomized | Unclear | 0 | 2 |
| Zhu, Y(2016) | Computer-generated random number tables | Only mentioned randomized | Single blinding | 0 | 4 |

**Table 2. Details of the Quality Assessment of Nocnrandomized Controlled Trial (Newcastle-Ottawa Scale)**

| Study | Study Design | Selection | Comparability | Exposure | Quality Assessment |
| --- | --- | --- | --- | --- | --- |
| Bai,Y. C(2013) | Concurrent retrospective chart review | 3 | 2 | 2 | 7 |
| Chaojie (2016) | Concurrent retrospective chart review | 3 | 2 | 2 | 7 |
| Deng, W(2014) | Concurrent retrospective chart review | 3 | 2 | 2 | 7 |
| Liu, Y (2018) | Concurrent retrospective chart review | 4 | 2 | 2 | 8 |
| Shen, H(2014) | Prospective controlled study | 4 | 2 | 2 | 8 |
| Shi, C (2016) | Prospective controlled study | 4 | 2 | 2 | 8 |
| Wang, B(2016) | Concurrent retrospective chart review | 4 | 2 | 1 | 7 |
| Fu, H (2017) | Prospective controlled study | 3 | 2 | 2 | 7 |
| Hao, R. T (2012) | Prospective controlled study | 4 | 2 | 2 | 8 |
| Su, A. P (2016) | Concurrent retrospective chart review | 4 | 2 | 1 | 7 |
| Wang, X. L(2009) | Prospective controlled study | 4 | 2 | 2 | 8 |
| Xue, S(2018) | Prospective controlled study | 4 | 2 | 1 | 7 |

**Table 3. The injection details of carbon nanoparticles used in the Meta-analysis**

| Study | Other Tracer | Injection points | Dose per point (mL) | Interval (min) |
| --- | --- | --- | --- | --- |
| \| Bai, Y. C (2013) \| \| --- \| \| Chaojie (2016) \| \| Chen, W (2014) \| \| Deng, W(2014) \| \| Fu, H (2017) \| \| Gao, B (2015) \| \| Gao, Q (2014) \| \| Gu, J(2015) \| \| Hao, R. T (2012) \| \| Liu, F (2017) \| \| Liu, Y (2018) \| \| Long, M(2017) \| \| Shen, H(2014) \| \| Shi, C (2016) \| \| Su, A. P (2016) \| \| Tian, W(2014) \| \| Wang, B(2015) \| \| Wang, B.(2016) \| \| Wang, X. L(2009) \| \| Xu, X. F (2017) \| \| Xue, S(2018) \| \| Yu, W (2016). \| \| Yu, WB(2016) \| \| Zhu, H(2017) \| \| Zhu,Y(2016) \| | Blank  Blank  Blank  MB  Blank  Blank  Blank  Blank  Blank  Blank  Blank  Blank  Blank  Blank  Blank  Blank  Blank  MB  Blank  Blank  Blank  Blank  Blank  Blank/MB  Blank | 2  2-3  3-4  1  3  2-4  3-4  ＜5  1  1  4  3  1  2  3  3  2  2  4-6  ＜5  4-6  2-3  2-3  2  1-2 | 0.1  0.1-0.15  0.2  1  0.1  0.2  0.1-0.2  0.1  0.1  0.1  0.1  0.1-0.2  0.1-0.2  0.1  0.2  0.05  0.1-.2  0.1-0.5  0.1  0.1  0.2*0.3  0.1-0.2  0.1–0.3  0.2  0.1 | 10-20  3-5  5-10  5  5  10-15  5-10  NM  5  5-10  30  5  3  10  10  20  20  NM  NM  10  1-5d  30  1-3  10-15  15 |
