# Supplementary material for: The role of carbon nanoparticle in lymph node detection and parathyroid gland protection during thyroidectomy for non-anaplastic thyroid carcinoma- a meta-analysis
Source: PLoS One. 2020 Nov 10;15(11):e0223627. doi: 10.1371/journal.pone.0223627 (PMC7654818; doi:10.1371/journal.pone.0223627)
Supplement: S2 File — (DOCX) [file pone.0223627.s003.docx]

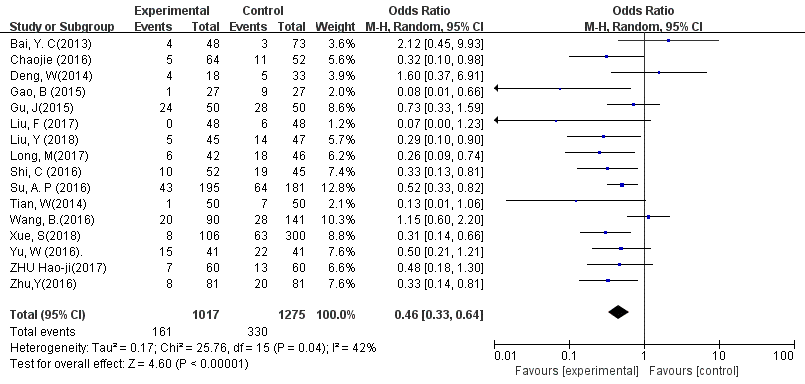


**Fig 1.** **Forest plots of the postoperative transient hypoparathyroidism rate. (Experimental=Carbon nanoparticle group, Control=Blank or methylene blue group, Total= The number of patient, Events= the number of patients who suffered from transient hypoparathyroidism. )**


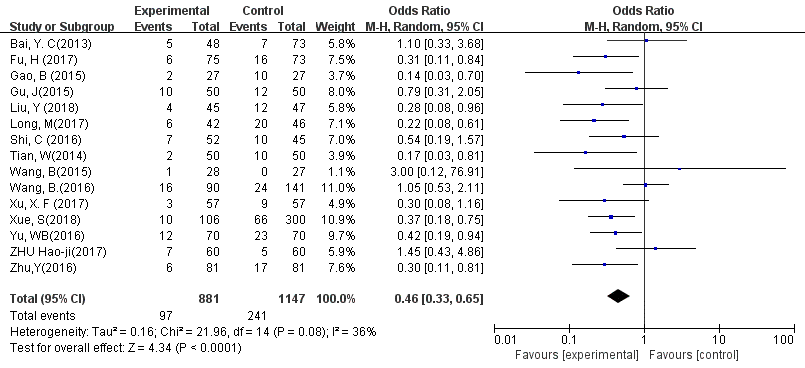


**Fig 2. Forest plots of the postoperative transient hypocalcemia rate. (Experimental=Carbon nanoparticle group, Control=Blank or methylene blue group, Total= The number of patient, Events= the number of patients who suffered from transient hypocalcemia. )**


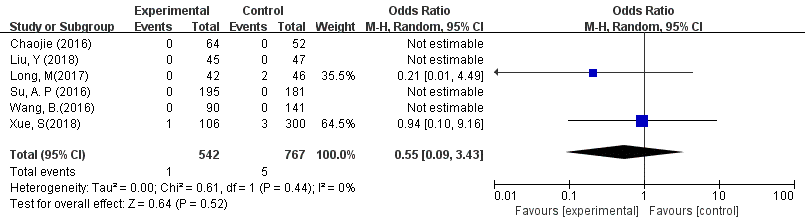


**Fig 3. Forest plots of tbe postoperative permanent hypoparathyroidism rate. (Experimental=Carbon nanoparticle group, Control=Blank or methylene blue group, Total= The number of patient, Events= the number of patients who suffered from permanent hypoparathyroidism. )**


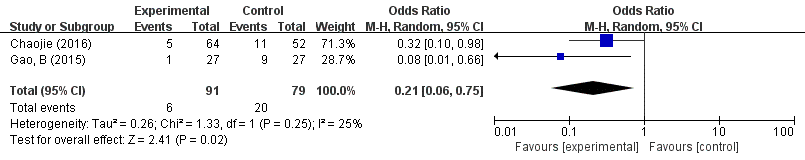


**Fig 4. Forest plots of the postoperative transient hypoparathyroidism during reoperation. (Experimental=Carbon nanoparticle group, Control=Blank or methylene blue group, Total= The number of patient, Events= the number of patients who suffered from transient hypoparathyroidism during reoperation.)**


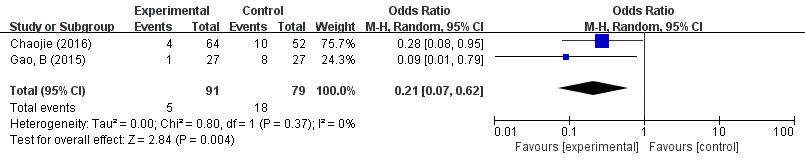


**Fig 5. Forest plots of the accidental parathyroid removal rate in groups during reoperation. (Experimental=Carbon nanoparticle group, Control=Blank or methylene blue group, Total= The number of patient, Events= the number of parathyroid glands removed accidentally during reoperation. )**
