# Supplementary material for: The role of carbon nanoparticle in lymph node detection and parathyroid gland protection during thyroidectomy for non-anaplastic thyroid carcinoma- a meta-analysis
Source: PLoS One. 2020 Nov 10;15(11):e0223627. doi: 10.1371/journal.pone.0223627 (PMC7654818; doi:10.1371/journal.pone.0223627)
Supplement: S3 File — (DOCX) [file pone.0223627.s004.docx]

1. <https://pubmed.ncbi.nlm.nih.gov/30620402/>
2. <https://pubmed.ncbi.nlm.nih.gov/26462967/>
3. <https://pubmed.ncbi.nlm.nih.gov/27741354/>
4. <https://pubmed.ncbi.nlm.nih.gov/24260876/>
5. <https://pubmed.ncbi.nlm.nih.gov/28267706/>
6. <https://pubmed.ncbi.nlm.nih.gov/26943721/>
7. <https://pubmed.ncbi.nlm.nih.gov/8702308/>
8. <https://pubmed.ncbi.nlm.nih.gov/10089947/>
9. <https://pubmed.ncbi.nlm.nih.gov/26309638/>
10. <https://pubmed.ncbi.nlm.nih.gov/32086925/>
11. <https://pubmed.ncbi.nlm.nih.gov/30198867/>
12. <https://pubmed.ncbi.nlm.nih.gov/25761552/>
13. <https://pubmed.ncbi.nlm.nih.gov/29095266/>
14. <https://pubmed.ncbi.nlm.nih.gov/25083465/>
15. <https://pubmed.ncbi.nlm.nih.gov/25897006/>
16. https://pubmed.ncbi.nlm.nih.gov/26799652/
17. <https://pubmed.ncbi.nlm.nih.gov/26783268/>
18. <https://pubmed.ncbi.nlm.nih.gov/25567436/>
19. <https://pubmed.ncbi.nlm.nih.gov/24330873/>
20. <https://pubmed.ncbi.nlm.nih.gov/30093623/>
21. <https://pubmed.ncbi.nlm.nih.gov/22521260/>
22. <https://pubmed.ncbi.nlm.nih.gov/29492083/>
23. <https://pubmed.ncbi.nlm.nih.gov/27759629/>
24. <https://pubmed.ncbi.nlm.nih.gov/27241116/>
25. <https://pubmed.ncbi.nlm.nih.gov/19558888/>
26. <https://pubmed.ncbi.nlm.nih.gov/26081253/>
27. <https://pubmed.ncbi.nlm.nih.gov/27861338/>
28. <https://pubmed.ncbi.nlm.nih.gov/25895310/>
29. <https://pubmed.ncbi.nlm.nih.gov/25567435/>
30. <https://pubmed.ncbi.nlm.nih.gov/25832013/>
31. <https://pubmed.ncbi.nlm.nih.gov/27634477/>
32. <https://pubmed.ncbi.nlm.nih.gov/25311844/>
33. <https://pubmed.ncbi.nlm.nih.gov/25895304/>
34. <https://pubmed.ncbi.nlm.nih.gov/26783035/>
35. <https://pubmed.ncbi.nlm.nih.gov/26885201/>
36. <https://pubmed.ncbi.nlm.nih.gov/27989915/>
37. <https://pubmed.ncbi.nlm.nih.gov/29798247/>
38. <https://pubmed.ncbi.nlm.nih.gov/29322020/>
