# Supplementary material for: The role of carbon nanoparticle in lymph node detection and parathyroid gland protection during thyroidectomy for non-anaplastic thyroid carcinoma- a meta-analysis
Source: PLoS One. 2020 Nov 10;15(11):e0223627. doi: 10.1371/journal.pone.0223627 (PMC7654818; doi:10.1371/journal.pone.0223627)
Supplement: Attachment — Submitted filename: Response to Reviewers.docx [file pone.0223627.s005.docx]

Dear reviewers,

Thank you for your review of our manuscript: PONE-D-19-26418 - “The Role of Carbon Nanoparticle in Lymph Node Detection and Parathyroid Gland Protection during Thyroidectomy- a Meta Analysis”. We really appreciate the reviewers’ valuable comments. We are resubmitting a revised manuscript, per their suggestions. We describe below the changes we have made as well as our response to the reviewer’s comments. The main changes were highlighted in red.

Journal Requirements:

1. When submitting your revision, we need you to address these additional requirements. Please ensure that your manuscript meets PLOS ONE's style requirements, including those for file naming.

Response:

The manuscript has been revised according to PLOS ONE's style requirements.

2. Please provide the full search strategy and full search terms used for at least one database used in the supplementary information. In addition, please provide additional details regarding the assessment of publication bias in your systematic review and meta-analysis. Specifically, please provide the funnel plots calculated from the Begg's test and/or the regression plots from the Egger's test.

Response:

The full search strategy and full search terms used has been listed in the Method section: page 4, line 69-72 . In addition, the funnel plots calculated from the Begg's test has been added in the text: page 7, line 125-126.

3. We note that you have stated that you will provide repository information for your data at acceptance. Should your manuscript be accepted for publication, we will hold it until you provide the relevant accession numbers or DOIs necessary to access your data. If you wish to make changes to your Data Availability statement, please describe these changes in your cover letter and we will update your Data Availability statement to reflect the information you provide.

Response:

Yes, I will provide the relevant information if needed.

Reviewers’ comments:

1. In addition, authors should note that their Figures 4-7 demonstrate that accidental parathyroid removal & hypo-parathyroidism is worse with CN (experimental) than with control. This is the opposite of what the authors report in the results and discussion and conclusion. Please explain. it is either a mistake in conclusion or a mistake in the way the treatment assignment variable direction is coded.

Response:

Thanks for your careful review. I made a mistake in the way the treatment assignment variable direction is coded, which have been corrected in the revised manuscript.(Modified at line 149, line 154, and line 163)

2. Also, authors should report the chance-corrected agreement (i.e. kappa) in the screening stages and the eventual final set of included studies.

Response:

The chance-corrected agreement has been added and inserted in page 6, line 117.

3. Please clarity the diagnosis included in the paper. Thyroid carcinoma is a varied and diverse group of diseases, ranging from anaplastic to papillary, which in the recent ATA guidelines has been downgraded in many cases. It needs to be clear which disease you are examining in the review.

Response:

Papillary thyroid carcinoma, follicular thyroid carcinoma, and medullary thyroid carcinoma comprising the subjects of the 25 studies included in the current meta-analysis. It’s impossible to distinguish them base on the data available. I combined these 3 pathologic types and termed as Non-anaplastic thyroid carcinoma, and make revision in the text where appropriate. The details were inserted in page 6, line 119-121.

4. Why did you only include studies that include single medical team/surgeon? This seems like an odd inclusion criteria.

Response:

I delete the criteria in manuscript. ( Modified in page 4, line 78-79.)

5. For your exclusion criteria, what did you include for "incomplete data"? You provide to description of what is considered complete data in your inclusion criteria. Do patients need to have follow up? Data on calcium levels? Of note, many of the included papers do not have simple data, like the average age. Why were these papers included while others were not?

Response:

Thanks for your critical review. “Incomplete data” is vague and inaccurate, so I deleted this exclusion criteria. (Modified in page 4, line 82-83.)

In addition, only 7 studies had a follow-up time of at least 6 months and they are eligible for the subgroup analysis of postoperative permanent hypocalcemia/hypoparathyroidism. I mentioned this in page 14, line 173.

6. In the abstract is it stated that only RCTs or non randomized CT are included however in the methods, it appears that retrospective trials were included. Can you please clarify this contradiction?

Response:

All the studies included in the meta-analsis were RCTs or Non-RCTs, and there was no retrospective trials included. I have corrected in the appropriate part of the text: page 5, line 98-101.

7. Please note the results in the figure diagrams are unclear. I cannot confirm the number of studies included or excluded and for which reasons based on the formatting errors.

Response:

The figures have been corrected and inserted in the appropriate place in the text.

8. The actual patient population and surgeries performed in the respective studies is unclear - how many were reoperations? How many were original surgeries? This is important data for the reader to be able to see and assess

Response:

I added the mentioned data in page 6, line 122-123.

9. Examining the results and figure, there is a high degree of variability in the actual number of studies included. I can't seem to find a single figure that includes all 25 included papers. Many of the sub analyses have small sample sizes (2 papers), which is quite different that the 25 papers reported in the results. Each sub analysis should be well described in the methods and results section, with a clearly states number or papers included and sample size for that analysis. Without this, the results of your paper are presented in a misleading way, implying there is more power to your analysis than their actual is

Response:

Thanks for your comment. In fact, studies included had different objectives and provided only their associate data. Therefore, there was no analysis including 25 studies. In addition, I add the data of number or papers included and sample size for each subgroup analysis according to your advice. (Modified at 112-185 )

10. I would suggest editing these figures and tables for consistent text format and readability. The content is cut off in several of the images. Additionally, there are far too many figures and tables, with scattered data throughout that is not easier for the reader to assess and interpret for themselves.

Answer: Thank you for your kindly advice. I summarized the result of each subgroup analysis in Table 2 for more intuitive.

We look forward to your acceptance.

Best regards,

Peng Hanwei.
