# Supplementary material for: The role of carbon nanoparticle in lymph node detection and parathyroid gland protection during thyroidectomy for non-anaplastic thyroid carcinoma- a meta-analysis
Source: PLoS One. 2020 Nov 10;15(11):e0223627. doi: 10.1371/journal.pone.0223627 (PMC7654818; doi:10.1371/journal.pone.0223627)
Supplement: Attachment — Submitted filename: Response to Reviewers.docx [file pone.0223627.s006.docx]

**Corresponding Author: Hanwei Peng**

**Affiliation:** Cancer Hospital of Shantou University Medical College

**Address:** No.7 Raoping Road, Shantou, Guangdong Province, China, 515031

**E-Mail: [penghanwei@126.com](mailto:penghanwei@126.com) Tel:** +86 13726510488

Dear reviewers,

Thank you for your review of our manuscript: PONE-D-19-26418 - “The Role of Carbon Nanoparticle in Lymph Node Detection and Parathyroid Gland Protection during Thyroidectomy- a Meta Analysis”. We really appreciate the reviewers’ valuable comments. We are resubmitting a revised manuscript, per their suggestions. We describe below the changes we have made as well as our responses to the reviewer’s comments. The main changes were highlighted in red.

1. Fig 1 is cut off. It needs to be fixed please.

Response:

We have fixed the Fig 1 in the manuscript. (Modified at line 124-125 )

2. Line 141-142 and Fig 3 are discordant: "WMD analysis showed that the total number of harvested lymph nodes in the CN group was significantly higher than that in the control groups" however Fig 3 shows the opposite. Please address this.

Response：

Thanks for your sharp review. We made a mistake in the way the treatment assignment variable direction is coded, which have been corrected in the revised manuscript. (Fig3, Modified at line 147)

3. Supplemental figures 1 and 2 also show the opposite of your conclusions; they show that control performs better whereas your conclusions state that experimental (CN) performs better.

Response：

We are so sorry that we made the same mistakes in our first revision in the Supplemental fig 1 and fig 2. We have revised these data and check throughout the manuscript to confirm that there is no similar mistakes remained.

4. Supplemental figure 3 and line 179-183: this is inappropriate use of fixed effects modeling. With only 2 studies dealing with heterogenous populations and also including comparisons with near-zero values, there is an unacceptably high risk of unstable estimates with fixed effects modeling. This should be done with random effects.

Response：

Thank you for your kindly advise. Random effects have been used in Supplemental figure 3, figure 4, figure5 and the related text in the manuscript have been revised. (See line 32-33, 170, 173-174, 177-180, Table2)

Thank you for your consideration of our revised manuscript. We look forward to the re-review of our manuscript and are prepared to make additional revisions as required.

Yours Faithfully,

Hanwei Peng
