# Supplementary material for: The role of carbon nanoparticle in lymph node detection and parathyroid gland protection during thyroidectomy for non-anaplastic thyroid carcinoma- a meta-analysis
Source: PLoS One. 2020 Nov 10;15(11):e0223627. doi: 10.1371/journal.pone.0223627 (PMC7654818; doi:10.1371/journal.pone.0223627)
Supplement: Attachment — Submitted filename: Response to Reviewers.docx [file pone.0223627.s007.docx]

**Corresponding Author: Hanwei Peng**

**Affiliation:** Cancer Hospital of Shantou University Medical College

**Address:** No.7 Raoping Road, Shantou, Guangdong Province, China, 515031

**E-Mail: [penghanwei@126.com](mailto:penghanwei@126.com) Tel:** +86 13726510488

Dear reviewers,

Thank you for your review of our manuscript: PONE-D-19-26418 - “The Role of Carbon Nanoparticle in Lymph Node Detection and Parathyroid Gland Protection during Thyroidectomy- a Meta Analysis”. We really appreciate the reviewers’ valuable comments. We are resubmitting a revised manuscript, per their suggestions. We describe below the changes we have made as well as our responses to the reviewer’s comments. The main changes were highlighted in red.

Q1: How exactly does using CN help identify parathyroid glands? Is it just that the parathyroid glands are not mistaken for lymph nodes since the lymph nodes would turn black? Are parathyroid glands commonly excised because they are thought to be lymph nodes or is it simply because they get stuck to the capsule? Please clarify specifically how the CN would help identify parathyroid glands.

Response: CN technique was described in detail in introdunction. (Modified at line 54-59)

Q2: Please include the pseudo confidence interval as part of the legend of Figure 2

Response: We describe the 95% confidence interval in results. (Modified at line 127)

Q3: There are a number of punctuation and grammatical errors throughout the manuscript, please review carefully and correct these

Response: Thanks for your sharp review. We made a mistake in punctuation and grammatical errors, which have been corrected in the revised manuscript. (Modified at line 108, 115-117, 138, 153, 160, 174, 184, 254)

Q4: Please clear the matter of the high percentages that you described as post surgical incidence of hypocalcemia and hypoparathyroidism: they are valid only in transient cases. Permanent hypocalcemia or hypoparathyroidism are described in the literature usually in less than 2% of cases.

Response: A more accurate expression was modified as your kindly advise. (Modified at line 46-47)

Q5: Please describe the CN technique more detailed.

Response: The same as Q1.

Q6: Please explain the reason for with you did consider only adolescents over 16 years old and adults in your study.

Response: To reduce the publication bias because most of the studies included only report the cases in adult or adolescents over 16 years old. (Modified at line 87-89)

Q7: Please define more clearly " Included patients with benign thyroid disease unable to be separated from N-ATC.

Response: Thank you for your kindly advise. We have modified it more clearly in exclusion criteria. (Modified at line 86-87)

Q8: Please describe the definition of transient respectively permanent hypocalcamie/hypoparathyroidism. Some studies use the PTH value less than 10 pg/mL, others less than 7 pg/mL. Some do state the permanent condition six months after the surgery, other use a different time interval. Please do comments which threshold and time interval were considered in the evaluated studies.

Response: We describe the definition of hypoparathyroidism and hypocalcemia in Discussion. (Modified at line 224-228)

Actually, most studies included in meta-analysis report the cases of hypoparathyroidism and hypocalcemia, but not the value of PTH in detail.

Thank you for your consideration of our revised manuscript. We look forward to the re-review of our manuscript and are prepared to make additional revisions as required.

Yours Faithfully,

Hanwei Peng
