# Supplementary material for: The role of carbon nanoparticle in lymph node detection and parathyroid gland protection during thyroidectomy for non-anaplastic thyroid carcinoma- a meta-analysis
Source: PLoS One. 2020 Nov 10;15(11):e0223627. doi: 10.1371/journal.pone.0223627 (PMC7654818; doi:10.1371/journal.pone.0223627)
Supplement: Attachment — Submitted filename: Response to Reviewers.docx [file pone.0223627.s008.docx]

**Corresponding Author: Hanwei Peng**

**Affiliation:** Cancer Hospital of Shantou University Medical College

**Address:** No.7 Raoping Road, Shantou, Guangdong Province, China, 515031

**E-Mail: [penghanwei@126.com](mailto:penghanwei@126.com) Tel:** +86 13726510488

Dear reviewers,

Thank you for your prompt re-review of our manuscript: PONE-D-19-26418 - *“The Role of Carbon Nanoparticle in Lymph Node Detection and Parathyroid Gland Protection during Thyroidectomy- a Meta Analysis”*. After discussing the comments raised by the reviewer, we made changes per the reviewers’ comments. We are now resubmitting a revised manuscript to correct the errors together with this cover letter for further evaluation.

Q1: Hypoparathyroidism definition is still not addressed.

**Response:** We addressed the hypoparathyroidism definition as well as diagnostic criteria in the current study in the manuscript. (See line 104-110)

Q2: If the CN helps identify more lymph nodes but does NOT help identify more diseased lymph nodes, is it worth utilizing? What would be the benefit of identifying and excising more benign lymph nodes?

**Response:** Thanks for your question. This is really a crucial point that readers may concern. In fact, in the *results* section, we stated " WMD analysis showed that the total number of harvested lymph nodes in the CN group was significantly higher than that in the control groups (WMD, 2.36; 95% CI, 1.40 to 3.32; *P*＜0.01, Fig 3).” (see line 157-159) and “No difference was found between the CNs group and the Control group regarding LN metastatic rate (OR = 1.07, 95% CI = 0.75 to 1.51, *P*=0.71, Fig 4).” (See line 163-164). These denoted that the numbers of both diseased lymph nodes and benign lymph nodes were higher in the CN group than that in the control group. Therefore, we advocate that this technique is worth utilizing. We revised a sentence in the discussion section (line 229-231) and added a sentence in this paragraph (line 234-237) to express our points of view.

Thank you for your consideration of our re-revised manuscript. We look forward to further review of our manuscript and are prepared to make additional revisions as required.

Yours Faithfully,

Hanwei Peng
